# A New Classification of Ectopic Pregnancies

**Published:** 1895-08-31

**Authors:** Charles Bodon

**Affiliations:** of Budapest


### Medical Progress and Hospital Clinics.

[*The Editor will be glad to receive offers of co-operation and contributions from members of the profession. All letters should be addressed to* The Editor, The Lodge, Porchester Square, London, W.]

### A NEW CLASSIFICATION OF ECTOPIC PREGNANCIES.

By Charles Bodon, M.D., of Budapest.

There is hardly any question which has been more discussed in recent times than that of ectopic pregnancies. The number of communications referring thereto is enormous, and the subject appears the more complicated as there are several points about it in regard to which the opinions of our greatest authorities are diametrically opposed. We find also that the authors of text-books, neglecting the opinions of others, like to advance their own. This is certainly a mistake in dealing with questions which are yet very far from being settled.

The old traditional classification of ectopic pregnancies into tubal, ovarian, and abdominal varieties proved to be too narrow. Lawson Tait's ingenious opinion that all ectopic pregnancies are primarily tubal, although supported by several authors, is not yet entirely proved, and far from being generally admitted.

Studying the literature of the subject I found *quot capita, tot sensus*, as many authors, as many classifications. This, of course, shows the difficulty of settling the question.

Experiences gained at the operating table are seldom of value in making out the nature of the case, because, in the hurry of the operation, it is often with these reports as, according to Goethe, it is with books, viz., we often find, not what is to be found, but what we wish to find.

I might illustrate this fact by a quite recent experience of mine. I assisted lately at an ovariotomy, which was performed by a well-known surgeon. The peritoneal cavity being opened, a large cyst, apparently ovarian, could be detected, which contained several hard masses. There escaped on tapping a reddish serous fluid, which had the peculiar smell of a macerated fœtus. The surgeon looked very pleased, and made the remark that he would not be astonished if it were a case of ovarian gestation. Well, he did not say "ectopic," but he said "ovarian" gestation, before he hardly knew anything about the relations of the tumour. No doubt he would have liked to get a case of ovarian pregnancy. He seemed disappointed, as it proved to be a parovarian cyst. I might be called a pessimist, but I am not quite sure whether the case, if the tumour happened to be a gestation-sac, would not have been published as a case of ovarian pregnancy.

As a matter of fact, we have chiefly to depend on observations where a careful post-mortem has been made, and where doubtful relations have been cleared up with the aid of a microscope. Unfortunately the number of such minute, exact records is yet relatively very small.

Another source of mistake lies in the fact that the classifications of ectopic pregnancy to be found in text-books are incomplete. The result is that many a practitioner, who has not made a particularly profound study of this question, will force his case, even if not quite suitable, into one of the varieties contained in his favourite handbook.

I think the latter inconvenience will be best checked by such a classification of extra-uterine pregnancy as shall extend to all possible forms already described, disregarding whether their occurrence has been conclusively proved by anatomical and histological research or not.

As such a classification I propose the following:—

**I. Primary Extra-Uterine Gestation.**—The ovum is actually in the same medium, where it got originally attached after fecundation.

Sub-varieties: (1) Tubo-uterine, or interstitial gestation: The ovum develops in that part of the tube which runs through the uterine parietes, which is called isthmus tubæ. (2) Tubo-abdominal gestation: The ovum develops in the free part of the tube. (3) Tubo-ovarian gestation: The ovum develops in a tubo-ovarian cyst. (4) Primary intraperitoneal gestation: The ovum gets attached in the free peritoneal cavity. (5) Ovarian gestation: The ovum is developed in the interior of the ovary. (6) Ovario-abdominal gestation: The ovum starts development on the surface of the ovary.

**II. Secondary and Tertiary Extra-Uterine Gestation.**—The ovum escapes after rupture of the sac from the medium, where it has been previously developing, and may go on living in the new surroundings.

Sub-varieties: (1) Secondary intraperitoneal gestation: The ovum escapes into the peritoneal cavity. (2) Extraperitoneal gestation: The ovum escapes into the connective tissue of the broad ligament which exists between its peritoneal folds. (3) Partial intraperitoneal gestation: May develop from the latter if one part of the ovum gets through a rent of the peritoneum into the peritoneal cavity. (4) Tertiary intraperitoneal gestation: May also develop from the extraperitoneal variety, if the ovum escapes later through a rent of the peritoneum entirely into the peritoneal cavity.

